# AllergoOncology – the impact of allergy in oncology: EAACI position paper

**DOI:** 10.1111/all.13119

**Published:** 2017-01-23

**Authors:** E. Jensen‐Jarolim, H. J. Bax, R. Bianchini, M. Capron, C. Corrigan, M. Castells, D. Dombrowicz, T. R. Daniels‐Wells, J. Fazekas, E. Fiebiger, S. Gatault, H. J. Gould, J. Janda, D. H. Josephs, P. Karagiannis, F. Levi‐Schaffer, A. Meshcheryakova, D. Mechtcheriakova, Y. Mekori, F. Mungenast, E. A. Nigro, M. L. Penichet, F. Redegeld, L. Saul, J. Singer, J. F. Spicer, A. G. Siccardi, E. Spillner, M. C. Turner, E. Untersmayr, L. Vangelista, S. N. Karagiannis

**Affiliations:** ^1^ The Interuniversity Messerli Research Institute University of Veterinary Medicine Vienna Medical University of Vienna Vienna Austria; ^2^ Institute of Pathophysiology & Allergy Research Center of Pathophysiology, Infectiology & Immunology Medical University Vienna Vienna Austria; ^3^ Division of Genetics & Molecular Medicine Faculty of Life Sciences and Medicine St. John's Institute of Dermatology King's College London London UK; ^4^ Division of Cancer Studies Faculty of Life Sciences & Medicine King's College London Guy's Hospital London UK; ^5^ LIRIC‐Unité Mixte de Recherche 995 INSERM Université de Lille 2 CHRU de Lille Lille France; ^6^ Division of Asthma, Allergy and Lung Biology Medical Research Council and Asthma UK Centre in Allergic Mechanisms in Asthma King's College London London UK; ^7^ Division of Rheumatology, Immunology and Allergy Department of Medicine Brigham and Women's Hospital Harvard Medical School Boston MA USA; ^8^ INSERM CHU Lille European Genomic Institute of Diabetes Institut Pasteur de Lille U1011 – récepteurs nucléaires, maladies cardiovasculaires et diabète Université de Lille Lille France; ^9^ Division of Surgical Oncology Department of Surgery David Geffen School of Medicine at UCLA Los Angeles CA USA; ^10^ Division of Gastroenterology, Hepatology and Nutrition Research Department of Medicine Research Children's University Hospital Boston Boston MA USA; ^11^ Randall Division of Cell and Molecular Biophysics King's College London London UK; ^12^ NIHR Biomedical Research Centre at Guy's and St. Thomas’ Hospitals and King's College London King's College London Guy's Hospital London UK; ^13^ Center Pigmod Institute of Animal Physiology and Genetics Academy of Sciences of Czech Republic Libechov Czech Republic; ^14^ Pharmacology and Experimental Therapeutics Unit Faculty of Medicine School of Pharmacy The Institute for Drug Research The Hebrew University of Jerusalem Jerusalem Israel; ^15^ Sackler Faculty of Medicine Tel‐Aviv University Tel‐Aviv Israel; ^16^ IRCCS San Raffaele Scientific Institute Milan Italy; ^17^ Department of Microbiology, Immunology, and Molecular Genetics David Geffen School of Medicine at UCLA Los Angeles CA USA; ^18^ Jonsson Comprehensive Cancer Center University of California Los Angeles CA USA; ^19^ Division of Pharmacology Faculty of Science Utrecht Institute for Pharmaceutical Sciences Utrecht University Utrecht The Netherlands; ^20^ Immunological Engineering Department of Engineering Aarhus University Aarhus Denmark; ^21^ ISGlobal Centre for Research in Environmental Epidemiology (CREAL) Barcelona Spain; ^22^ Universitat Pompeu Fabra (UPF) Barcelona Spain; ^23^ CIBER Epidemiología y Salud Pública (CIBERESP) Madrid Spain; ^24^ McLaughlin Centre for Population Health Risk Assessment University of Ottawa Ottawa ON Canada; ^25^ Department of Biomedical Sciences Nazarbayev University School of Medicine Astana Kazakhstan

**Keywords:** AllergoOncology, allergy, atopy, cancer, tumor, IgE, IgG4, chemotherapeutic, biologics, desensitization, clinical oncology, inflammation

## Abstract

Th2 immunity and allergic immune surveillance play critical roles in host responses to pathogens, parasites and allergens. Numerous studies have reported significant links between Th2 responses and cancer, including insights into the functions of IgE antibodies and associated effector cells in both antitumour immune surveillance and therapy. The interdisciplinary field of AllergoOncology was given Task Force status by the European Academy of Allergy and Clinical Immunology in 2014. Affiliated expert groups focus on the interface between allergic responses and cancer, applied to immune surveillance, immunomodulation and the functions of IgE‐mediated immune responses against cancer, to derive novel insights into more effective treatments. Coincident with rapid expansion in clinical application of cancer immunotherapies, here we review the current state‐of‐the‐art and future translational opportunities, as well as challenges in this relatively new field. Recent developments include improved understanding of Th2 antibodies, intratumoral innate allergy effector cells and mediators, IgE‐mediated tumour antigen cross‐presentation by dendritic cells, as well as immunotherapeutic strategies such as vaccines and recombinant antibodies, and finally, the management of allergy in daily clinical oncology. Shedding light on the crosstalk between allergic response and cancer is paving the way for new avenues of treatment.

## Graphical abstract

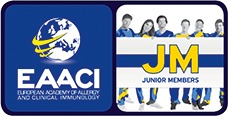

It has been recognized that tumours manipulate immune responses. On the other hand, the overall immune competence of the host could critically determine immune surveillance against cancer and the clinical course. Allergy and atopy are characterized by a systemic bias to Th2 immunity, which may exert a potential influence on cancer development. In fact, allergy and oncology may represent two opposite concepts: whereas immune tolerance is desired in allergy, it is detrimental in cancer. Hence, the establishment of a Task Force on AllergoOncology (AO) within the Immunology Section of EAACI is timely and appropriate. The aim of the Task Force is to connect basic scientists interested in Th2 immunity and cancer with clinical oncologists and to support an interdisciplinary exchange to advance knowledge and understanding of immune responses in both fields. At present, this is the first AO platform worldwide.

Previous AO activities have included a first concerted paper 1, international conferences, the book ‘IgE and Cancer’ 2 and a Symposium‐in‐Writing on AO 3.

A Pre‐Task Force Meeting was held at the EAACI annual conference in Copenhagen in 2014, leading to the establishment of the Task Force within the Interest Group of Immunology, with its first business meeting in Barcelona in 2015. The primary objectives of the Task Force were confirmed: to serve as an interface between the disciplines of oncology and allergy, covering: (i) basic, (ii) translational, (iii) epidemiological and (iv) clinical research, including allergy problems in clinical oncology, as well as (v) mechanisms of tumour‐induced immune modulation and (vi) novel vaccination and immunotherapy approaches harnessing IgE functions to target cancer.

The goal of this position paper was to provide an update on developments in the AO field since 2008 1. We therefore aimed to review (i) clinical, mechanistic and epidemiological insights into Th2 immune responses in cancer, (ii) current immunological markers with a complementary role in allergy and cancer, (iii) correlation of these markers with the progress of malignant diseases and (iv) an update on how oncologists can manage allergic reactions to cancer therapeutics.

The different topics were drafted by subgroups of the Task Force and further discussed, developed and compiled during a meeting in Vienna in 2015. The position paper was thereafter recirculated and critically appraised, and the final version was approved by all Task Force members.

## Epidemiology

The epidemiologic association between allergy and cancer risk has been summarized in meta‐analyses, with inverse associations reported for several cancers including glioma, pancreatic cancer, and childhood leukaemia 4, 5. The majority of previous studies have relied on self‐reported ascertainment of allergic status, being typically limited, retrospective, and associated with potential biases. Emerging evidence comes from prospective studies based on self‐reported allergy history which have reported inverse associations in studies of colorectal 6, but not haematopoietic or prostate cancer 7, 8. A large‐scale study based on hospital discharge records reported an inverse association between allergy/atopy of at least 10 years in duration and incidence of brain cancer [RR (Relative Risk) = 0.6, 95% CI (Confidence Interval) 0.4–0.9] in a cohort of 4.5 million men 9. Nested case–control studies reported inverse associations between borderline or elevated total IgE 10 or respiratory‐specific IgE and glioma risk 10, 11. Serum total and allergen‐specific IgE provided evidence of inverse associations with the development of melanoma, female breast cancer, gynaecological malignancies and also glioma 12. Findings at other cancer sites are unclear 13, 14, 15. One study reported an inverse trend between increasing blood eosinophil count and subsequent colorectal cancer risk 16. Another study reported that serum concentrations of soluble CD23/FcεRII (sCD23) and soluble CD30 (sCD30) were positively associated with risk of non‐Hodgkin's lymphoma 17. Several studies have examined associations with SNPs in allergy‐related genes with significant associations between SNPs in *FCER1A, IL10, ADAM33, NOS1* and *IL4R* genes and glioma risk reported in one recent study which requires further replication 18.

Further research in large‐scale prospective studies using validated measures of self‐reported allergy history and/or biomarkers of allergy is needed, including repeated evaluations over time, sufficient latency with respect to the developing tumour, and detailed data on potentially confounding variables 19.

## Th2‐associated antibodies in cancer

Although studied for decades, our understanding of different immunoglobulin classes in cancer biology is still limited. IgG antibodies are the predominant antibody class for passive immunotherapy. Recent findings elucidated that the tumour microenvironment may specifically promote less potent immunoglobulin isotypes such as IgG4 20. Furthermore, IgG and IgE free light chains engaging mast cells could reduce tumour development *in vivo* 21. Furthermore, by promoting specific phenotypes of tumour infiltrating leucocytes and through inducing a higher expression of inhibitory Fcγ receptors, malignant cells can evade humoral immune responses and counteract the antitumour effector functions of therapeutic IgG antibodies 22.

A preliminary study has reported that both IgE and IgG4 specific towards two of three cancer antigens are elevated in patients with cancer compared with healthy volunteers 23. The phenomenon that anticancer therapies, such as alkylating agents and hormone‐based chemotherapies, affect circulating total and specific IgE levels has also been reported 24; however, any implications on clinical course require further investigations. A number of these studies also provide evidence in support of Th2 humoral immunity to derive new tools for malignant disease monitoring and prognosis. Interestingly, IgE antibodies isolated from patients with pancreatic cancer mediate antibody‐dependent cell‐mediated cytotoxicity (ADCC) against cancer cells 25. Furthermore, higher levels of polyclonal IgE in nonallergic individuals are directly correlated with lower disease incidence and higher survival in multiple myeloma in a clinical study 26. Collectively, these studies point to important roles for Th2‐associated antibodies and tumour immune surveillance.

## *In situ* expression of AID and potential insights into antibody isotype expression in cancer

The enzyme cytidine deaminase (AID) which is responsible for converting cytidine to uracil and thereby induces targeted damage to DNA, is a key driver of immunoglobulin (Ig) somatic hypermutation events and class switch recombination processes that give rise to IgG, IgA or IgE. On the other hand, AID has multifaceted functions linking immunity, inflammation and cancer 27.

AID is thought to be expressed predominantly by germinal centre (GC) B cells within secondary lymphoid organs. However, studies on local autoimmunity, transplant rejection, and tissues exposed to chronic inflammation point to the capacity of B lymphocytes to form GC‐like ectopic structures outside of secondary lymphoid tissues 27, 28, which is now also demonstrated within benign and malignant tissues. Class switching of local GC‐derived B cells to different isotypes may have a profound influence on local immune responses and on disease pathobiology. However, whether tumour microenvironments support direct class switching to IgE remains unclear, although some evidence from animal models points to IgE production at early stages of carcinogenesis 29. Remarkably, local follicle‐driven B cell‐attributed immune responses may be either positively or negatively associated with clinical outcomes of patients with cancer 30, 31.

## IgE receptor expression on immune cells and epithelial cells

**Figure 1:**
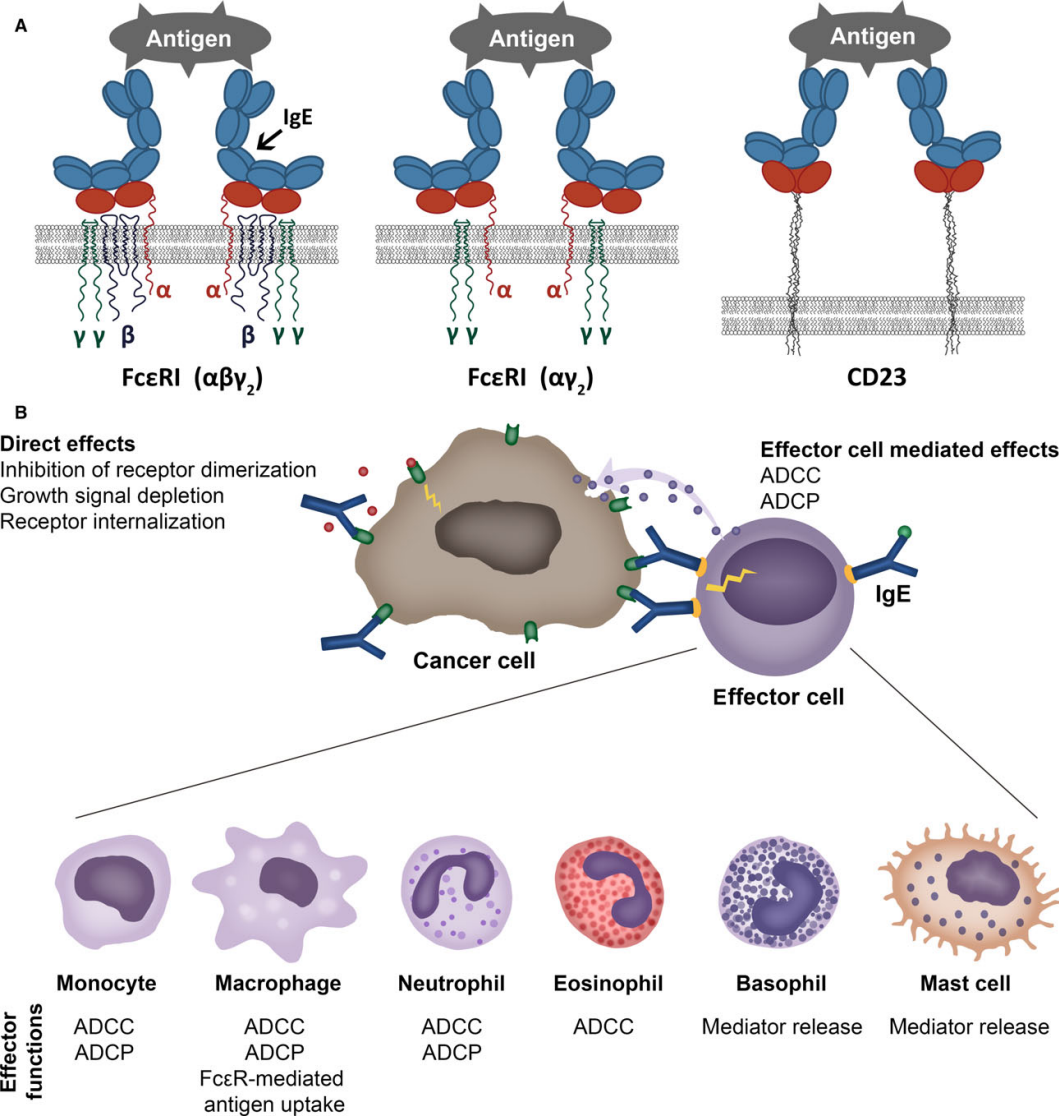
Cell surface IgE receptors and IgE‐mediated direct and indirect effects. (A) Cartoon of IgE binding to its cell surface receptors. IgE binds to tetrameric (αβγ_2_) (left) and trimeric forms (αγ_2_) (middle) of FcεRI through the extracellular domain of the alpha (α) chain of the receptor. The low‐affinity receptor CD23 trimer binds IgE through recognition of the lectin domain (right). (B) Direct and cell‐mediated effects of antitumour IgE. Like IgG antibody therapies, IgE targeting tumour antigens can exert direct effects through recognizing the target antigen, such as interference with signalling, resulting in growth inhibition. IgE can also bind via IgE receptors (FcεRI or FcεRII/CD23) to a specific repertoire of effector cells (illustrated in the bottom panel). These interactions may lead to effector functions against tumour cells, such as antibody‐dependent cell‐mediated phagocytosis (ADCP) or cytotoxicity (ADCC), or mediator release. Cross‐linking of IgE is required for effector cell activation, whereas soluble tumour antigens expressing only a single epitope do not trigger IgE cross‐linking on the surface of effector cells.

The high‐affinity receptor FcεRI tetrameric form αβγ2 is expressed on mast cells and basophils. The trimeric form of the high‐affinity receptor FcεRI (αγ2) and the low‐affinity receptor CD23/FcεRII (b form) (Fig. 1A) is expressed on human monocytes and macrophages, dendritic cells (DCs), eosinophils, platelets and neutrophils 32. The ‘a’ form of CD23/FcεRII is also expressed by subsets of B cells 33. IgE cell surface receptors FcεRI, FcεRII/CD23 (Fig. 1A) and also the soluble IgE receptors galectin‐3 and galectin‐9 are expressed not only by haematopoietic cells, but also by nonhaematopoietic cells including epithelia (Table 1).

**Table 1:** Expression of IgE‐binding structures on haematopoietic or nonhaematopoietic cells in humans

IgE‐binding structure	Receptor composition/splice variants	Expression on haematopoietic cells	Expression on nonhaematopoietic cells
High‐affinity IgE receptor/FcεRI	Tetrameric receptor αβγ_2_	Mast cells, basophils Kraft S, Kinet JP. *Nat Rev Immunol* 2007 May;7(5):365–78	–
	Trimeric receptor αγ_2_	Mast cells, basophils Kraft S, Kinet JP. *Nat Rev Immunol* 2007;7(5):365–78 Monocytes, macrophages Boltz‐Nitulescu G, et al. *Monogr Allergy* 1983;18:160–2 Spiegelberg HL. Int *Rev Immunol* 1987;2(1):63–74 Dendritic cells Novak N, et al. *J Clin Invest* 2003; 111(7):1047–56 Bieber T, et al. *J Exp Med* 1992; 175(5):1285–90 Allam JP, et al. *J Allergy Clin Immunol* 2003; 112(1):141–8 Bannert C, et al. *PLoS One* 2012**;** 7(7):e42066 Yen EH, et al. *J Pediatr Gastroenterol Nutr* 2010**;** 51(5):584–92 Eosinophils Gounni AS et al. *Nature* 1994, 367(6459):183–6 Platelets Hasegawa S et al. *Blood* 1999; 93(8):2543–51.	Small intestinal and colonic epithelial cells Untersmayr et al. *PLoS One* 2010 Feb 2;5(2):e9023
	α chain	Neutrophils Dehlink et al., *PLoS One* 2010, 5(8):e12204 Alphonse MP et al. *PLOS One* 2008; 3(4):e1921	Paneth cells Untersmayr E et al. *PLoS One* 2010;5(2):e9023 Smooth muscle cells Gounni AS et al. *J Immunol* 2005;175(4):2613–21
Low‐affinity IgE receptor/FcεRII/CD23	CD23a isoform	Antigen‐activated B cells Reviewed in: Gould HJ, Sutton BJ. *Nat Rev Immunol* 2008;8(3):205–17. doi: 10.1038/nri2273	
	CD23b isoform	B cells Yukawa K, et al. *J Immunol* 1987;138(8):2576–80 Monocytes, macrophages Vercelli D et al. *J Exp Med* 1988;167(4):1406–16 Pforte A et al. *J Exp Med* 1990;171(4):1163–9 Eosinophils Capron M et al. *Chem Immunol* 1989;47:128–78 Platelets Capron M et al. *J Exp Med* 1986;164(1):72–89 Dendritic cells Bieber T et al. *J Exp Med* 1989;170(1):309–14	Small intestinal and colonic epithelial cells Kaiserlian D et al. *Immunology* 1993 Sep;80(1):90–5
Galectins	Galectin‐3	Monocytes, macrophages Liu FT et al. *Am J Pathol* 1995;147(4):1016–28 Neutrophils Truong MJ, et al. *J Exp Med* 1993;177(1):243–8 Eosinophils Truong MJ et al. *Eur J Immunol* 1993;23(12):3230–5 Basophils and mast cells Craig SS et al. *Anat Rec* 1995;242(2):211–9 Dendritic cells Brustmann H. *Int J Gynecol Pathol* 2006;25(1):30–7 Smetana K et al. *J Leukoc Biol* 1999;66(4):644–9	Gastric cells Fowler M et al. *Cell Microbiol* 2006;8(1):44–54 Small intestinal, colonic, corneal, conjunctival and olfactory epithelial cells, epithelial cells of kidney, lung, thymus, breast, prostate Dumic J, et al. *Biochim Biophys Acta* 2006;1760(4):616–35 Jensen‐Jarolim E et al. *Eur J Gastroenterology & Hepatol* 2002;14(2):145–52 Uterine epithelial cells von Wolff M et al. *Mol Hum Reprod* 2005;11(3):189–94 Fibroblasts Openo KP et al. *Exp Cell Res* 2000;255(2):278–90 Chondrocytes and osteoblasts Janelle‐Montcalm A et al. *Arthritis Res Ther* 2007;9(1):R20 Osteoclasts Nakajima K et al. *Cancer Res* 2016;76(6):1391–402 Keratinocytes Konstantinov KN et al. *Exp Dermatol* 1994;3(1):9–16 Neural cells Pesheva P et al. *J Neurosci Res* 1998; 54(5):639–54
	Galectin‐9	T cells Chabot S et al. *Glycobiology* 2002 Feb;12(2):111–8 Monocytic cells, macrophages Harwood NM et al. *J Leukoc Biol* 2016; 99(3):495–503 Mast cells Wiener Z et al. *J Invest Dermatol* 2007; 127(4):906–14	Intestinal epithelial cells Chen X et al. *Allergy* 2011;66(8):1038–46 M cells Pielage JF et al. *Int J Biochem Cell Biol* 2007;39(10):1886–901 Nasal polyp fibroblasts Park WS et al. *Biochem Biophys Res Commun* 2011;411(2):259–64 Endometrial epithelial cells Shimizu Y et al. *Endocr J* 2008;55(5):879–87 Endothelial cells Imaizumi T et al. *J Leukoc Biol* 2002; 72(3):486–91

Depending on the nature and distribution of IgE receptors, different functions might be envisaged. Galectin‐3 is well recognized for its contribution to tumour progression and metastasis development 34, while galectin‐9 seems to have antiproliferative effects 35, 36. The trimeric FcεRI(αγ2) showed membranous and cytoplasmic expression in intestinal epithelial cells and a prominent FcεRI α‐chain expression was also found in the Paneth cells of patients with cancer of the proximal colon. In the same study, a similar distribution could be observed in tissues from patients with gastrointestinal inflammation, whereas no expression was observed in healthy controls 37.

It is important to note that cell surface‐expressed IgE‐binding structures may have different effector functions compared with their secreted forms such as soluble FcεRIα chain 38 and galectin‐3 39 in cancer, which may be of key functional importance.

## Effector cells in allergy and cancer

### Mast cells

Mast cells are perhaps the most classical effector cells of IgE (Fig. 1B). Their presence at the periphery, but also infiltrating tumours, argues for a role in tumour biology 40. The presence of mast cells in many tumours has been associated with poor prognosis 41, and it has been suggested that they may contribute to an immunosuppressive tumour microenvironment and thereby impede protective antitumour immunity. In addition, mast cells may promote tumour growth by inducing angiogenesis and tissue remodelling through the induction of changes in composition of the extracellular matrix 42. In contrast, in colorectal cancers, mesothelioma, breast cancer, large B‐cell lymphoma, and in non‐small‐cell lung cancer, high mast cell density has been associated with favourable prognoses 43, 44. The observation of degranulating mast cells near dying tumour cells has suggested a cytotoxic effect and their presence in invasive breast carcinomas correlate with better prognosis 45. In prostate cancer, peri‐tumoral mast cells were shown to promote, while intratumoral mast cells may restrict angiogenesis and tumour growth 46. This apparent dichotomy in mast cell functions in cancer may be explained by (i) tumour type, (ii) tumour stage, (iii) mast cell phenotypic plasticity and (iv) location of mast cells in relation to tumour cells.

A wealth of evidence from human cancers and mouse models of cancer indicates that mast cells via the action of histamine on H1, H2 and H4 receptors contribute to tumour invasion and angiogenesis 44. Mast cells may also suppress the development of protective antitumour immune responses by promoting regulatory T cell (Treg)‐mediated suppression in the tumour microenvironment 47.

Mast cells are attracted to the tumour microenvironment by stem cell factor (SCF) secreted by tumour cells, and secrete pro‐angiogenic factors as well as matrix metalloproteinases (MMPs), which promote tumour vascularization and invasiveness. Stem cell factor is the ligand for CD117 (c‐kit receptor), highly expressed by mast cells. SCF is essential in mast cell recruitment, tumour‐associated inflammation, remodelling and immunosuppression 48. Stem cell factor‐stimulated mast cells produce matrix metalloprotease‐9 (MMP‐9) that facilitates recruitment of mast cells and other cells to the tumour. MMP‐9 also augments tumour‐derived SCF production in an amplification feedback loop. Using mast cell‐deficient (C57BL/6‐Kit^W‐sh/W‐sh^) mice, it was shown that mast cells (and mast cell‐derived MMP‐9) are necessary and sufficient to promote growth of subcutaneously engrafted prostate adenocarcinoma cells 49. Furthermore, mast cell tumour‐promoting potential is augmented through costimulation with tumour‐derived SCF and Toll‐like receptor 4 (TLR4) ligand, inhibiting mast cell degranulation, but triggering their production and secretion of vascular endothelial growth factor (VEGF) and interleukin‐10 (IL‐10). In contrast, mast cell stimulation by TLR4 ligand alone induces IL‐12, important regulator of T‐ and NK‐cell responses 50.

Fibroblast growth factor‐2 (FGF‐2) and VEGF derived from mast cells trigger intense angiogenic responses *in vivo* 46. Infiltration of mast cells and activation of MMP‐9 parallel the angiogenic switch in premalignant lesions and, accordingly, accumulation of mast cells is usually found in the proximity of CD31+ cells and microvessels 51. Mast cells are a major source of interleukin‐17 (IL‐17) which enhances microvessel formation, being negatively prognostic in gastric cancer 52. Mast cells may also contribute to an immunosuppressive tumour microenvironment as they mobilize the infiltration of myeloid‐derived suppressor cells (MDSCs) into tumours and induce the production of IL‐17 by MDSCs 47, which indirectly attracts Tregs, enhancing their suppressor function and IL‐9 production; in turn, IL‐9 strengthens the survival and pro‐tumour effect of intratumoral mast cells.

There is some evidence, however, that these pro‐tumoral activities of mast cells may be subverted by targeting these cells to promote tumour destruction. In a mouse allograft model, triggering of degranulation of mast cells by IgE antibody cross‐linking of cell surface FcεRI resulted in Treg cell impairment and acute CD4+ and CD8+ T cell‐mediated tissue destruction 53. In addition, human mast cells have been demonstrated to directly induce lymphoma tumour cell death *in vitro* when incubated with an anti‐CD20 IgE antibody 54. These insights suggest the potential to reactivate these cells against cancer through immunotherapies.

### Eosinophils

Blood and tissue eosinophilia are prominent features of allergy and also found to be associated with various cancers. Tumour‐associated eosinophilia (TATE) has been reported to correlate with good or with bad prognosis. Epidemiological and clinical studies suggest evidence of intratumoral eosinophil degranulation and tumoricidal activity 55 (Fig. 1B). Human eosinophils have been reported to induce colon cancer cell death *in vitro*, implying mechanisms involving innate receptors (TCRγδ/CD3 complex, TLR2) and mediators such as alpha‐defensins, TNF‐α, granzyme A and IL‐18 56, 57, 58. Tumoricidal functions of eosinophils were target antigen‐specific and differed among individuals. Additionally, tumour antigen‐specific IgE has been shown to trigger eosinophil‐mediated tumour cell death by cytotoxic mechanisms 59. Importantly, eosinophils from allergic donors proved more cytotoxic 56, which suggests that the allergic state favours antitumour processes.

### Macrophages

Tumour‐associated macrophages (TAMs) differentiate from monocyte precursors circulating in blood (Fig. 1B) and are recruited to tumour sites by several pro‐inflammatory molecules such as chemokines (C‐C motif chemokine ligand) CCL2, CCL3, CCL4, CCL5, and also VEGF, transforming growth factor‐β (TGF‐β) and colony‐stimulating factors (GM‐CSF and M‐CSF) 60. Even if their phenotype is under the control of specific tumour‐derived chemokines and cytokines that polarize macrophages to a pro‐immune ‘M1’ or immunosuppressive/pro‐angiogenic ‘M2’ phenotype, their transcriptional profiles are distinct from regular M1 or M2 macrophages 61. Tumour‐associated macrophages are characterized by high expression of CCL2, CCL5, and IL‐10 and by MGL1, dectin‐1, CD81, VEGF‐A, CD163, CD68, CD206, arginase‐1 (Arg‐1), nitric oxide synthase 2 (NOS2), MHC‐II and scavenger receptor A 62. Tumour‐associated macrophages may differ considerably in terms of function and M1/M2 phenotype, depending on the type of tumour, stage of progression and location within the tumour tissue 60, 63; The M1/M2 TAM heterogeneity could explain the poor prognosis in glioma and breast cancers and better prognosis in stomach and colon, prostate and non‐small‐cell lung cancers 60. Tumour‐associated macrophage heterogeneity depends on the localization in the tumour microenvironment: In normoxic areas, TAMs show a CD206^low^MHCII^hi^ M1‐like phenotype; in hypoxic areas TAMs show a CD206^hi^MHCII^low^ M2‐like phenotype 61, 63. The expression of Arg‐1 as well as VEGF‐A, Solute Carrier Family 2 members 1 and 3 (SCL2A1 and SCL2A3) and NOS2 are specifically modulated in hypoxic area in CD206^hi^MHCII^low^ TAMs 63. Innovative drugs allow the positive effects of elevating M1/kill and other anticancer innate responses but they also increase an undesired, ‘overzealous M1/kill–Th1 cytotoxic response’ contributing to chronic inflammation 64.

New strategies aim to re‐educate TAMs to exert antitumour functions. In fact, even in a Th2‐M2 tumour microenvironment macrophages stimulated by IL‐4 and IL‐13 were able to inhibit proliferation of B16‐F1 melanoma cells 65. Moreover, macrophages may via IgE and IgG binding to diverse receptors on them acquire anti‐tumour‐killing potency. Tumour‐associated macrophages express these receptors as well, enabling therapeutic monoclonal antibodies to engage in antibody‐dependent cell‐mediated cytotoxicity/phagocytosis (ADCC/ADCP) 66. Recent discoveries show IgG4‐positive cells in several tumour environments, possibly being attracted by CCL1–CCR8 interactions. The only macrophage subtype producing CCL1 is M2b which support vascularization and promote Th2‐biased tumour microenvironments 67. More research on IgG/IgE effector functions by TAMs is necessary to define new therapeutic concepts.

### Dendritic cells

**Figure 2:**
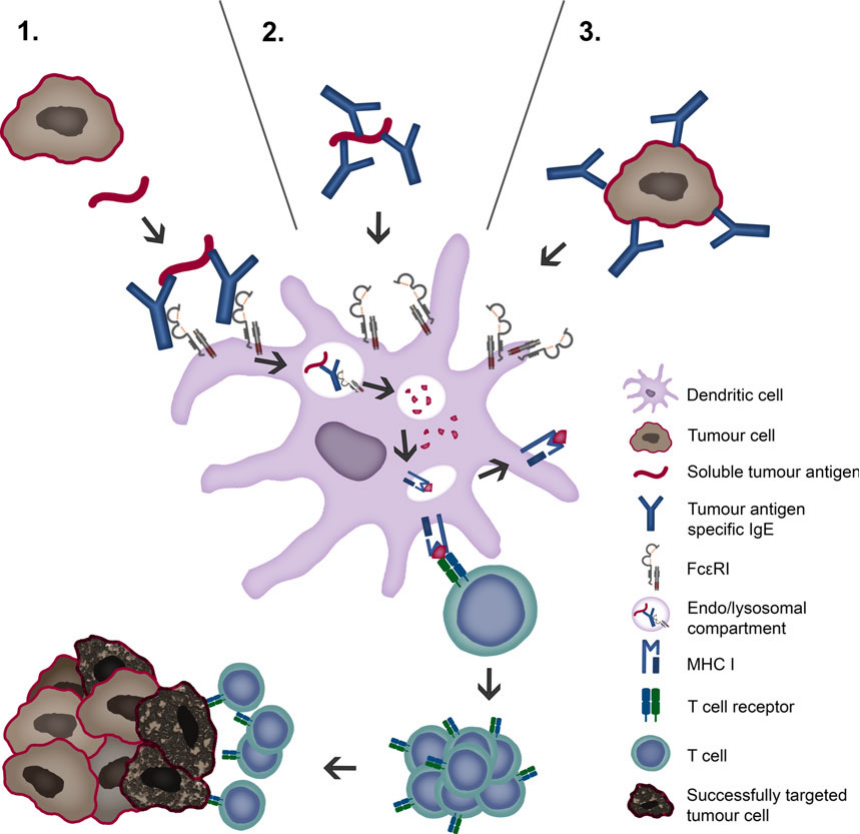
Tumour antigen uptake and presentation by dendritic cells recruits cytotoxic CD8+ T lymphocytes. Tumour cells display tumour antigens at a high density, facilitating cross‐linking of IgE fixed to FcεRI receptors on antigen‐presenting cells, such as dendritic cells (DCs). Tumour antigens may be taken up via three possible routes: (1) soluble tumour antigen binding to receptor‐bound IgE; (2) By IgE‐opsonized soluble antigen binding to IgE receptors and (3) IgE‐opsonized tumour cells binding to IgE receptors. Endocytosis of IgE–antigen complexes leads to digestion in lysosomes and loading of antigenic peptides on MHC I molecules. Cross‐presentation via proteasome, loading to MHC I and recognition by CD8+ T lymphocytes (CTLs) is depicted.

Antigen cross‐presentation by DCs is key feature of antitumour immunity as it results in the generation of cytotoxic CD8+ T lymphocytes (CTLs) against tumour antigens. Recently, an IgE‐mediated cross‐presentation pathway has been discovered 68, 69 (Fig. 2), resulting in priming of CTLs to soluble antigen at unusually low dose, and independent MyD88 signals or IL‐12 production by DCs. Passive immunization experiments and DC‐based vaccination strategies confirmed that IgE‐mediated cross‐presentation significantly improves antitumour immunity and even induces memory responses *in vivo*. However, IL‐4, a signature Th2 cytokine, efficiently blunted IgE‐mediated cross‐presentation indicative for a feedback mechanism that prevents overshooting CTL responses during allergy 70. Deciphering details of IgE/FcεRI‐mediated cross‐presentation will further provide new insights into the role of Th2 immune responses in tumour defence and improve DC‐based vaccination strategies.

### T lymphocytes

CD8+ T lymphocytes and Th1 cells play the central role in elimination of tumour cells by the immune system (Fig. 2). Th1 cells produce interferon‐gamma (IFN‐γ) which mediates antitumour activity by several mechanisms, including activation of macrophages, enhancement of antigen processing and presentation, and inhibition of angiogenesis 71. The role of Th2 cells in cancer is more controversial. In some cancers, including breast 72, gastric 73 and pancreatic cancer 74, 75, Th2 cells and associated cytokines [IL‐4, IL‐13, Thymic stromal lymphopoietin (TSLP)] contribute to tumour progression. In addition, IL‐4 plays a crucial role in the survival of colon cancer stem cells 76. Therefore, IL‐4 and IL‐13 receptors are promising anticancer targets 77. On the other hand, Th2 cells and cytokines can also play protective roles against cancer. In Hodgkin lymphoma, high numbers of Th2 cells are associated with better survival 78. Thymic stromal lymphopoietin has been shown to block early breast carcinogenesis through the induction of Th2 cells 79. Thymic stromal lymphopoietin can inhibit colon cancer by inducing apoptosis of cancer cells 80. Thymic stromal lymphopoietin and Th2 cells also mediate resistance to carcinogenesis in mice with epidermal barrier defect 81, 82.

Further, DCs pulsed with anti‐prostate‐specific antigen (PSA) IgE or with anti‐HER2/*neu* IgE antibody, complexed with antigen, induced enhanced CD4 and CD8 T‐cell activation *in vitro* compared to antigen‐complexed IgG1 83, 84. Similar observations were made upon OVA‐specific IgE/FcεRI‐mediated cross‐priming in DC and T‐cell cocultures, where CD8 T‐cell proliferation and granzyme B secretion were increased 69. Collectively, these findings support potential roles for Th2 responses in IgE immune surveillance against cancer.

## Translational strategies to target cancer

### Tumour vaccines and adjuvants

Different approaches to induce IgE‐mediated adaptive immunity against cancer have been designed.

A cellular vaccine based on tumour cells infected with modified vaccinia virus Ankara (MVA) and loaded with IgE conferred protection in mice upon tumour challenge, slower tumour growth and increased survival 85. This antitumour adjuvant effect may depend on the interaction of IgE with FcεRI as it was lost in FcεRIα^−/−^ mice, but not in CD23^−/−^ mice. In parallel, using a humanized hFcεRIα mouse model expressing the human FcεRIα chain, human IgE could exert antitumour adjuvant effects 86. When a human truncated mIgE (tmIgE) which retained binding to FcεRI and triggering immune cell activation was inserted into a rMVA, the resulting rMVA‐tmIgE showed a protective effect in the above humanized FcεRIα mouse model 86.

Other anticancer vaccine approaches are based on specific tumour antigens and tumour antigenic mimotopes which have shown promise in restricting tumour growth 87. When using evolutionarily conserved cancer antigens, such as HER2/*neu* or EGFR, a vaccine may be used across different species 88. Furthermore, the formulation with adjuvants like aluminium hydroxide, orally sucralfate or proton pump inhibitors, may help to direct induction of protective IgE antibody and merit careful study 89.

### Recombinant IgE anticancer antibodies

**Figure 3:**
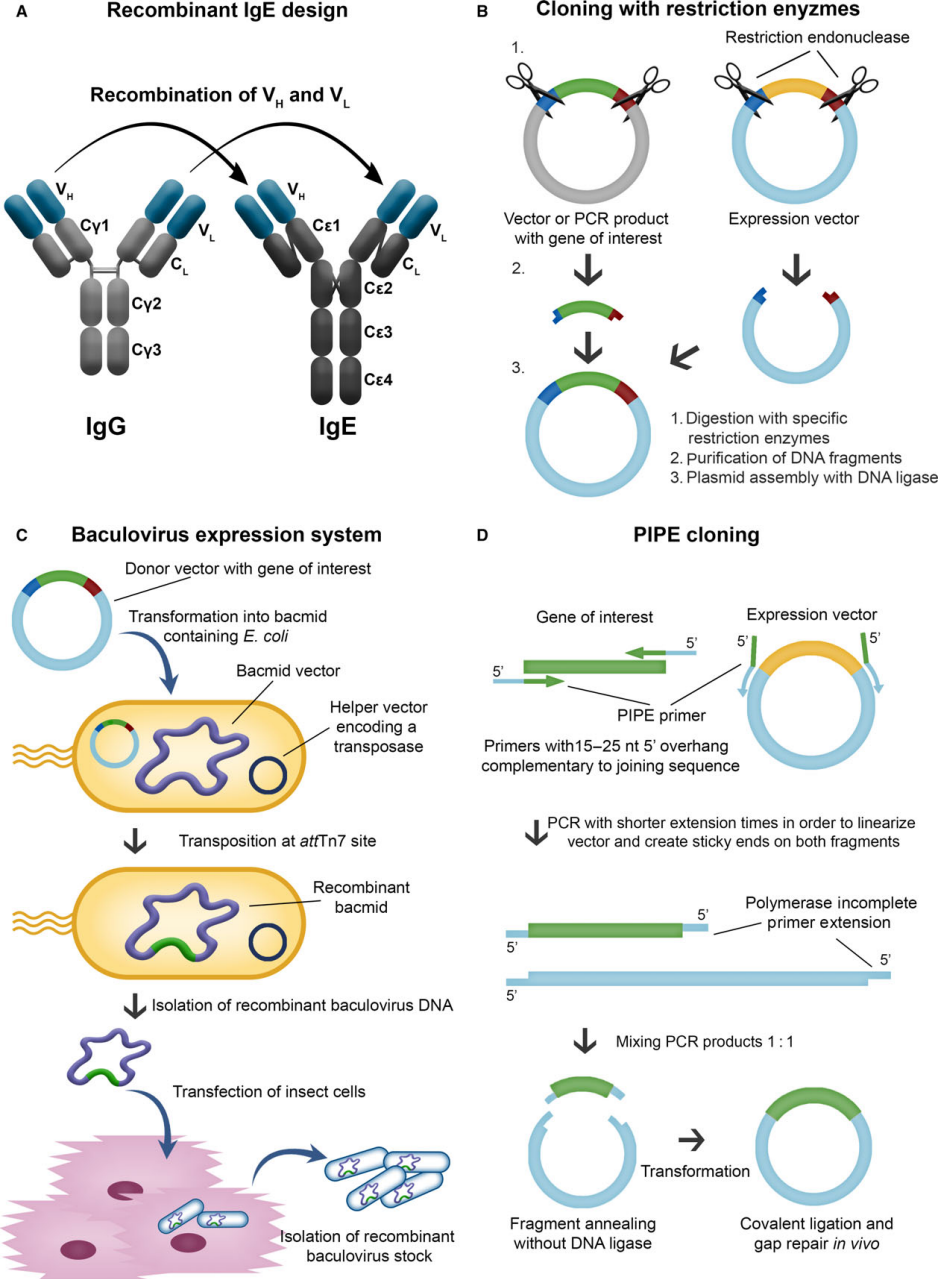
Examples of expression systems used for recombinant expression of antitumour IgE. (A) Recombinant IgE by cloning the variable domains of IgG of desired specificity to an IgE constant domain. (B) Classical restriction enzyme‐based cloning requires the presence of specific restriction sites flanking the gene of interest. (C) Expression of IgE by insect cells requires a recombinant baculovirus stock containing the antibody expression cassette. (D) Polymerase Incomplete Primer Extension (PIPE) cloning facilitates a rapid cloning of DNA sequences with the option of performing site‐specific mutagenesis at the same time.

Engineering antibodies with Fc regions of the IgE class specific for cancer antigens is designed to (i) harness the high affinity of this antibody isotype for its cognate Fcε receptors on tissue‐resident and potentially tumour‐resident immune effector cells and (ii) utilize the properties of IgE to exert immune surveillance in Th2 conditions such as in tumour microenvironments. Recombinant antibody technologies and approaches to recombinant IgE (rIgE) production have advanced significantly with a number of antibodies already engineered and tested *in vitro* and *in vivo* (see Table 2). Recombinant IgE can be generated by different cloning strategies (Fig. 3). While classical restriction enzyme‐based cloning requires the presence of specific restriction sites flanking the gene of interest, expression of IgE by insect cells requires a recombinant baculovirus stock containing the antibody expression cassette. Novel protocols enable site‐specific transposition of the coding sequence using bacmid‐containing *E. coli* as intermediate hosts. Polymerase Incomplete Primer Extension cloning, independent of restriction or other recombination sites, facilitates rapid cloning with the option of site‐specific mutagenesis at the same time. Human/mouse chimaeric IgEs were also generated by hybridoma technology from a knock‐in mouse strain 90, and more efficient cloning strategies using mammalian expression vectors are available 91. In future fully human IgE antibodies could be generated from synthetic human antibody repertoire libraries 92, or cloned directly from the B cells of patients 93.

**Table 2:** IgE antibodies targeting cancer antigens

IgE species	IgE specificity	Nomenclature	Technology used for production	Expression system	*In vitro* results	Route of IgE *in vivo* administration	Targeted cancer cells (route of cell inoculation)	Mouse model	References
Passive immunotherapy studies									
Mouse	gp36 of MMTV	Clone A8 and H11	Murine hybridoma	Fusion of spleen cells with P3X20 myeloma cells	NR	i.p.	H2712 mouse mammary carcinoma (s.c. and i.p.)	C3H/HeJ	Nagy E et al., Cancer *Immunol Immunother*. 1991;34(1):63–69.
Rat/human chimaeric	Mouse Ly‐2	YTS169.4	Genetic engineering	Murine hybridoma YTS169.4L	ADCC mediated by murine T cells expressing chimaeric FcεRI	s.c.	E3 mouse thymoma (s.c.)	C57BL/6	Kershaw MH et al., *J Leukoc Biol*. 1996;60(6):721–728.
Mouse and mouse/human chimaeric	Colorectal cancer antigen	mIgE 30.6 and chIgE 30.6	Genetic engineering	Murine myeloma (Sp2/0)	Antigen binding affinity	i.v.	Human COLO 205 (s.c.)	SCID	Kershaw MH et al., *Oncol Res*. 1998;10(3):133–142.
Rat/human chimaeric	Mouse Ly‐2	YTS169.4	Genetic engineering	Murine hybridoma YTS169.4L	ADCC mediated by human T cells expressing chimaeric FcRεI	i.p.	E3 mouse thymoma (i.p.)	NOD‐SCID	Teng MW et al., *Hum Gene Ther*. 2006;17(11):1134‐1143.
Mouse/human chimaeric	FBP	MOv18IgE	Genetic engineering	Murine myeloma (Sp2/0‐Ag14)	Degranulation and ADCC cytotoxicity mediated by platelets	i.v.	IGROV1 human ovarian carcinoma cells (s.c.)	C.B‐17 SCID/SCID	Gould HJ et al., *Eur J Immunol*. 1999;29(11):3527–3537.
					ADCC and ADCP mediated by human monocytes, ADCC mediated by human eosinophils	i.p.	HUA patient‐derived ovarian carcinoma (i.p.)	nu/nu	Karagiannis et al. 59
Humanized	HER2/*neu*	Trastuzumab IgE	Genetic engineering	HEK293	Antigen binding affinity, degranulation, ADCC, interaction with human monocytes, and direct cytotoxicity in human breast cancer cells	NR	NR	NR	Karagiannis et al. 99
Human	HER2/*neu*	C6MH3‐B1 IgE	Genetic engineering	Murine myeloma (P3X63Ag8.653)	Degranulation and IgE‐facilitated antigen stimulation	i.p.	D2F2/E2 mouse mammary carcinoma cells expressing human HER2/*neu* (i.p.)^a^	Human FcεRIα Tg BALB/c	Daniels et al. 83, 106
Mouse/human chimaeric	EGFR	425 IgE and 225IgE	Genetic engineering	HEK293	Direct cytotoxicity induced by 425 IgE, ADCC mediated by 225 IgE and human monocytes	NR	NR	NR	Spillner et al. 100
Mouse/human chimaeric	MUC1	3C6.hIgE	Genetic engineering	CHO‐K1	NR	s.c.	4T1 tumour cells expressing human MUC1 (s.c.)	Human FcεRIα Tg BALB/c	Teo et al. 54
Mouse/human chimaeric	CD20	1F5.hIgE	Genetic engineering	CHO‐K1	ADCC using human mast cells and eosinophils as effector cells	NR	NR	NR	Teo et al. 54
Vaccination studies									
Mouse	DNP	mAb SP6	Murine hybridoma	Murine hybridoma	NT	i.p.	MC38 mouse colon carcinoma cells expressing human CEA (s.c.)^b^	C57BL/6	Reali E et al., *Cancer Res*. 2001 15;61(14):5517–5522.
Mouse	DNP	SPE7	Murine hybridoma	Murine hybridoma	Degranulation of haptenized cells	s.c.	TS/A‐LACK mouse mammary carcinoma cells coated with DNP (s.c.)	BALB/c	Nigro et al. 85
Mouse/human chimaeric	NIP	Anti‐NIP IgE	Genetic engineering	J558L murine myeloma	Degranulation of haptenized cells	s.c.	TS/A‐LACK mouse mammary carcinoma cells coated with NIP (s.c.)	Human FcεRIα Tg BALB/c	Nigro et al. 85
Human (truncated)	N/A	tmIgE	Genetic engineering	Chicken embryo fibroblasts	Degranulation	s.c.	TS/A‐LACK mouse mammary carcinoma cells coated with truncated IgE (s.c.)	Human FcεRIα Tg BALB/c	Nigro et al. 86
Mouse/human chimaeric	PSA	Anti‐PSA IgE	Genetic engineering	Murine myeloma (Sp2/0‐Ag14)	Degranulation and IgE‐facilitated antigen stimulation	s.c.	CT26 tumour cells expressing human PSA (s.c.)	Human FcεRIα Tg BALB/c	Daniels‐Wells et al. 84

ADCC/ADCP, antibody‐dependent cell‐mediated cytotoxicity/phagocytosis; DNP, dinitrophenol (hapten); EGFR, epidermal growth factor receptor; FBP, folate binding protein; HEK, human embryonic kidney; HER2/*neu*, human EGFR2/neuroblastoma; i.p., intraperitoneal; i.v., intravenous; MMTV, mouse mammary tumour virus; MUC1, mucin‐1, cell surface‐associated; NIP, nitrophenylacetyl (hapten); NR, not reported; PSA, prostate‐specific antigen; s.c., subcutaneous; Tg, transgenic; SCID, severe combined immunodeficient.

a A pilot toxicity study also conducted in nonhuman primates (cynomolgus monkeys).

b Tumour targeting occurred via a biotinylated anti‐CEA IgG followed by streptavidin and then a biotinylated IgE.

Also the heavily glycosylated structure of the IgE antibody class has to be considered. IgE has seven glycosylation sites, six of which are occupied mainly by complex *N*‐glycans including terminal galactose, sialic acid and fucose structures 94. Oligomannosidic structures are only identified at position Asn394 94, and some evidence suggests that they may be involved in binding to IgE Fc receptors and in some biological activities of IgE 95. On the other hand, complex *N*‐glycosylation of IgE is not thought to have a direct impact on its ability to bind to FcεRI or CD23. The contribution of glycosylation on IgE binding to galectin‐3 and galectin‐9 96 remains unclear. There is increasing evidence that the glycosylation of IgEs in healthy and different disease states may vary, prompting the need for further research on the importance of glycans on IgE functions against cancer.

### *In vitro* effector functions of IgE antibodies in the cancer context

Eosinophil, monocyte and macrophage‐mediated ADCC/ADCP, antigen presentation by DCs and degranulation of mast cells and basophils, have been identified as potent mechanisms of IgE‐mediated anticancer functions *in vitro* 69, 97, 98 (Fig. 1B).

The ADCC/ADCP functions against cancer of the anti‐folate receptor‐alpha (FRα) IgE, MOv18, have been previously described 59. Recently, the therapeutic anti‐HER2/*neu* and anti‐EGFR IgG1 antibodies, trastuzumab (Herceptin^®^, Roche Diagnostics, Basel, Switzerland) and cetuximab (Erbitux^®^, Merck Biopharma, Darmstadt, Germany), respectively, have been cloned and engineered recombinantly as humanized and chimaeric IgE antibodies 99, 100. Using U937 monocytic effector cells 101, trastuzumab IgG1 mediated killing of HER2‐overexpressing CT26 murine as well as of SKBR3 human mammary carcinoma cells mainly by ADCP, whereas trastuzumab IgE mediated killing via ADCC 99. Similarly, cetuximab IgG1 and IgE mediated comparable levels of phagocytosis of EGFR‐overexpressing A431 human tumour cells by purified human monocytes, but cetuximab IgE triggered significantly higher levels of ADCC than IgG1 100. Eosinophil ADCC killing has also been demonstrated *in vitro* using an anti‐human CD20 IgE antibody against OCl‐Ly8 lymphoma cells 54. Incubation with this anti‐CD20 IgE stimulated cord blood‐derived mast cells to release IL‐8 and kill CD20+ tumour cells. Similarly, trastuzumab IgE, cetuximab IgE, anti‐PSA IgE, and MOv18 IgE activated RBL SX‐38 rat basophilic leukaemia cells when cross‐linked with anti‐IgE antibody engaged with multimeric antigen, or when incubated with target cells overexpressing specific tumour‐associated antigen 84, 99, 100, 102. In contrast, monomeric soluble tumour antigen did not trigger degranulation 84, 100, 102. Accordingly, incubation of MOv18 IgE‐sensitized RBL SX‐38 cells with patient sera containing elevated levels of soluble FRα, did not lead to mast cell degranulation. Furthermore, MOv18 IgE did not trigger the basophil activation in the presence of soluble FRα, which is highly elevated in the sera of subsets of ovarian carcinoma and mesothelioma patients 102. This suggests that in tumours, mast cells may release potent inflammatory mediators in the presence of tumour‐specific IgE and overexpressed tumour antigen, while IgE in the absence of multimeric antigen is not expected to trigger anaphylactic responses. Notably, antigenic epitopes also need to be displayed in a rigid spacing to lead to productive triggering 103. Given that the potent anticancer functions of IgE antibodies *in vitro,* it is important to consider these results in the patient context 104. The impact of soluble tumour antigen in the circulation has been considered from a safety perspective 97, 102. However, the possible inhibitory activity of soluble tumour antigen sequestering IgE and preventing tumour cell engagement with effector cells needs to be elucidated.

Furthermore, the ability of patient immune effector cells to eradicate malignant cells must be evaluated to consider: (i) the impact of treatments as chemotherapy or steroid intake on effector cell functions, (ii) the effect of the tumour microenvironment on IgE receptor expression and on killing properties of these effector cells, and (iii) whether IgE immunotherapy may itself re‐educate effector cells to enhance their antitumour functions 105.

### *In vivo* models in AllergoOncology

Various animal models have been successfully employed to study the *in vivo* efficacy of IgE antibodies against cancer 106, with different limitations.

Direct application of human IgE in tumour bearing mice is not applicable as human IgE does not bind rodent Fcε receptors (Table 3). Next, humans express FcεRI on a broad range of cells including monocytes, mast cells, basophils, eosinophils, platelets, Langerhans cells and DCs (Table 1), whereas murine FcεRI expression has only been confirmed on mast cells and basophils (Table 4). Furthermore, also CD23 can be found on numerous human cells, while mice express CD23 on only B cells and certain T cells 106.

**Table 3:** Cross‐reactivity of IgE and Fcε receptors of different species, with the equilibrium association constant (*K*_A_) or equilibrium dissociation constant (*K*_D_), where available, described exactly as in the original references

Species	Human FcεRI	NHP* FcεRI	Mouse FcεRI	Rat FcεRI	Dog FcεRI
Human IgE	*K*_A_ a = 0.5–2.7 × 10^9^ M^−1^ Ishizaka T, Soto CS, Ishizaka K. Mechanisms of passive sensitization. III. *J Immunol* 1973; 111(2):500–11 Pruzansky JJ, Patterson R. *Immunology* 1986; 58(2):257–62	*K*_D_ a = 1.876 × 10^−8^ M Saul L et al. *MAbs* 2014; 6(2):509–22	No binding Fung‐Leung WP et al. *J Exp Med* 1996;183:49–56	No binding Fung‐Leung WP et al. *J Exp Med* 1996;183:49–56	No binding Lowenthal M, Patterson R, Harris KE. *Ann Allergy* 1993; 71(5):481–4
NHP IgE	*K*_D_ = 3 × 10^−10^ M Meng YG, Singh N, Wong WL. *Mol Immunol* 1996; 33(7–8):635–42	N.D.	N.D.	N.D.	No binding Lowenthal M, Patterson R, Harris KE. *Ann Allergy* 1993; 71(5):481–4
Mouse IgE	*K*_A_ a = 4.4 × 10^8^ M^−1^	N.D.	*K*_A_ a = 1.75–3.57^b^ × 10^9^ M^−1^ Sterk AR, Ishizaka T. *J Immunol* 1982; 128(2):838–43	*K*_A_ a = 2.49–5.05^c^ × 10^9^ M^−1^ Sterk AR, Ishizaka T. *J Immunol* 1982; 128(2):838–43	N.D.
Rat IgE	*K*_D_ a = 1.58 × 10^−8^ M Mallamaci MA et al. *J Biol Chem* 1993; 268(29):22076–83	N.D.	*K*_A_ a = 1.46–2.68^b^ × 10^9^ M^−1^ Sterk AR, Ishizaka T. *J Immunol* 1982; 128(2):838–43	*K*_A_ a = 7.84–8.05^c^ × 10^9^ M^−1^ Sterk AR, Ishizaka T. *J Immunol* 1982; 128(2):838–43	N.D.
Dog IgE	*K*_D_ = 9.2 × 10^−9^ M Fung‐Leung WP et al. *J Exp Med* 1996;183:49–56 Ye H et al. *Mol Immunol* 2014; 57(2):151–9	Confirmed binding Fung‐Leung WP et al. *J Exp Med* 1996;183:49–56	N.D.	N.D.	*K*_D_ = 2.1 × 10^−8^ M Ye H et al. *Mol Immunol* 2014; 57(2):151–9

N.D., not determined; NHP, non‐human primates.

a Affinity determination based on cells, not receptor subunits; therefore, also CD23 binding might contribute to the denoted values.

b Depending on mouse strain used as a source of mast cells.

c Depending on mast cell source (rat or RBL cell line).

**Table 4:** Tissue distribution of IgE receptors in humans *vs* animal models in AllergoOncology

**Human**	Basophils, mast cells, eosinophils, monocytes, dendritic cells, Langerhans cells Reviewed in: Daniels TR et al. *Cancer Immunol Immunother* 2012;61(9):1535–1546	Monocytes, eosinophils, B cells, T cells, dendritic cells, Langerhans cells, platelets Rev. in: Daniels TR et al. *Cancer Immunol Immunother* 2012;61(9):1535–1546	
**Mouse (wt)**	Basophils, mast cells Rev. in: Daniels TR et al. *Cancer Immunol Immunother* 2012;61(9):1535–1546	μ+, δ+ B cells, some CD8+ T‐cell subsets Delespesse G et al. *Immunol Rev* 1992 Feb;125:77–97	
**Mouse (transgenic)**			
*mFc*ε*RI*α^*−/−*^ *, hFc*ε*RI*α*Tg, C57BL/6 background* Dombrowicz D et al. *Immunity* 1998;8(4):517–529	hFcεRIα on bone marrow‐derived mast cells Dombrowicz D et al. *J Immunol* 1996 Aug 15;157(4):1645–51		mFcεRIα is replaced with hFcεRIα, which complexes with murine FcRβ and FcRγ subunits
*hFc*ε*RI*α*Tg, C57BL/6J background* Fung‐Leung WP et al. *J Exp Med* 1996 Jan 1; 183(1): 49–56	hFcεRIα on bone marrow‐derived mast cells Fung‐Leung WP et al. *J Exp Med* 1996 Jan 1; 183(1): 49–56. PMCID: PMC2192401		hFcεRIα complexes with murine FcRβ and FcRγ subunits
*Model 1: mFc*ε*RI*α^*−/−*^ *, hFc*ε*RI*α*Tg* *Model 2: mFc*ε*RI*α^*−/−*^ *, mFcR*β^*−/−*^ *, hFc*ε*RI*α*Tg, BALB/c background* Dombrowicz D et al. *Immunity*, Volume 8, Issue 4, 1 April 1998, Pages 517–529	Mast cells, basophils, monocytes, eosinophils, Langerhans cells Dombrowicz D et al. *Immunity*, Volume 8, Issue 4, 1 April 1998, Pages 517–529		mFcεRIα is replaced with hFcεRIα, which complexes with murine FcRβ and/or FcRγ subunits
**Rat**	Basophils, mast cells, macrophages, eosinophils Daniels TR et al. *Cancer Immunol Immunother* 2012;61(9):1535–1546	B cells, macrophages Capron A et al. *Eur J Immunol* 1977 May;7(5):315–22 Mencia‐Huerta JM et al. *Int Arch Allergy Appl Immunol* 1991;94(1–4):295–8	
**Dog**	Basophils, tissue mast cells, monocytes, Langerhans cells, CD1+ dendritic cells Jackson HA et al. *Veterinary Immunology and Immunopathology*, Volume 85, Issues 3–4, March 2002, Pages 225–232	Eosinophils Galkowska H et al. *Veterinary Immunology and Immunopathology*, Vol 53, Issues 3–4, October 1996, Pages 329–334	Data not complete, sometimes not evident if expression relates to FcεRI or CD23

Despite these differences, a xenograft model with severe combined immunodeficient (SCID) mice, and a patient‐derived xenograft model of ovarian carcinoma both reconstituted with human peripheral blood mononuclear cells, were successfully used to demonstrate the superior tumour‐killing potential of MOv18 IgE over IgG1 via both CD23 and FcεRI 59, 107. More human‐relevant models have been established using transgenic mice strains that express human FcεRIα, which complexes with endogenous murine FcRβ and FcRγ subunits, forming fully functional tetrameric FcεRI on mast cells and possibly trimeric receptors on macrophages, Langerhans cells and eosinophils, with a tissue distribution like in humans 108, 109, 110. A human anti‐HER2/*neu* (C6MH3‐B1 IgE) IgE tested in this model significantly prolonged the survival of immunocompetent mice bearing HER2/*neu*‐expressing tumours 83. A constraint of these transgenic models is the lack of CD23 expression, precluding evaluation of IgE‐triggered CD23‐mediated phagocytosis 111. A surrogate immunocompetent model system of syngeneic carcinoma in rats aims to better recapitulate the human IgE immune system 105, as FcεRI and CD23 expression and cellular distribution in rat cells, including monocytes and macrophages, mirrors that of humans.

Relevant models to address toxicity of human IgE antibodies are nonhuman primates (NHP) such as cynomolgus (*Macaca fascicularis*) and rhesus (*Macaca mulatta*) monkeys, as they have been shown to mediate anaphylaxis induced by human IgE 112. Cynomolgus monkeys have been routinely used to evaluate the safety of IgG therapeutic antibodies currently used in the clinic. A fully human IgE antibody targeting HER2/*neu* (C6MH3‐B1 IgE), administered systemically, was also well tolerated in cynomolgus monkeys 83. However, while recent studies confirmed the cross‐reactivity of human IgE with cynomolgus monkey peripheral blood leucocytes (PBLs) with comparable binding kinetics, human IgE dissociates faster from cynomolgus monkey PBLs and triggers a different cytokine release profile 113. This is important in toxicity studies using IgE in this species. Further, also differences in the interaction of FcγRs with the various human IgG isotypes have been found 114. Thus, while NHP are meaningful models for toxicity studies of human antibodies, they must be used with caution.

The dog (*Canis lupus familiaris*) is another potential model, as dogs suffer from spontaneous cancer and atopic diseases, making them a relevant clinical experimental model 115. For instance, the canine counterparts to HER2/*neu* and canine EGFR expression are highly homologous to the human molecules, and can be targeted by trastuzumab and cetuximab 88. An additional advantage is the remarkable similarity of the human and dog immune systems in terms of immunoglobulin classes, IgE and FcεRI expression and functional homology 116. Thus, canine anti‐EGFR IgG has been generated 117 and IgE is currently being generated for comparative studies in canine cancer patients.

The described animal models are informative for preclinical testing, but clinical trials in human patients are required to fully understand the therapeutic potential and risks associated with IgE anticancer antibodies.

## Allergy in clinical oncology: a cross‐disciplinary field

Allergic reactions to anticancer drugs are a common clinical problem, seen especially with platinum drugs, taxanes, anthracyclines 118 and monoclonal antibodies (mAbs) 119. In some cases, it is the excipient rather than the drug itself that is responsible for the hypersensitivity reaction. The administration of some of these drugs is routinely preceded by premedication with steroids.

**Figure 4:**
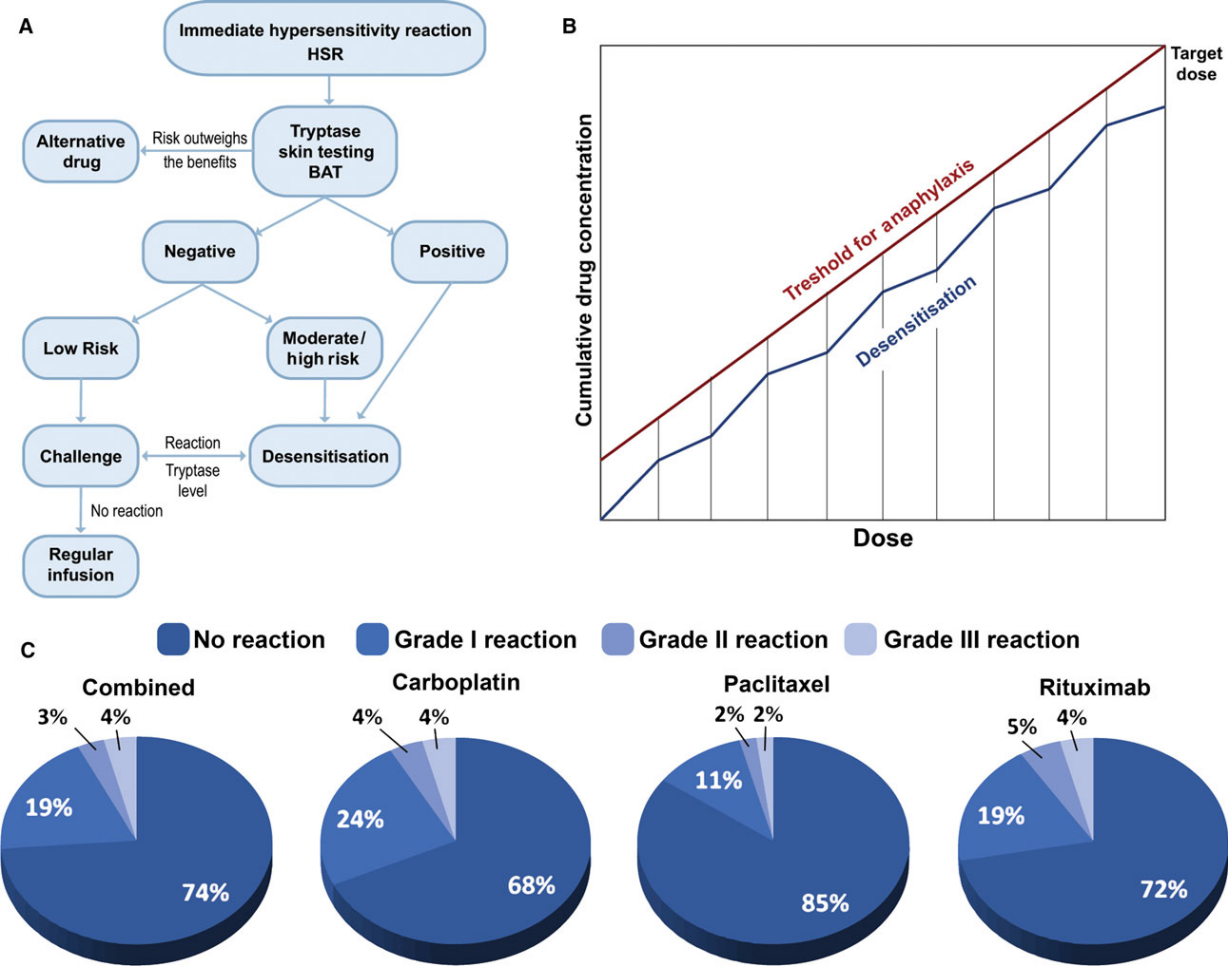
Treating hypersensitivity in clinical oncology. (A) Proposed algorithm for the evaluation of hypersensitivity of chemotherapy drugs and indications for rapid drug desensitization (RDD). BAT, basophil activation test; HSR, immediate hypersensitivity reaction. (B) Proposed mechanism for chemotherapy RDD (adapted from Ref. 164). (C) Outcomes of Brigham and Women's Hospital desensitization protocols for carboplatin, paclitaxel and rituximab in 2177 cases for 370 patients (adapted from Ref. 133).

Treatment of allergic anticancer drug reactions is, as for other episodes of hypersensitivity, using intravenous fluids, antihistamines, steroids and antipyretics, depending on the risk evaluation (Fig. 4A). Desensitization algorithms can be used in cases of established drug allergy, in which escalating small doses of the drug are administered in a controlled environment with ready access to critical care facilities 120 (Table 5). Desensitization has a particular role to play in clinical scenarios where repeated rechallenge with an active drug may be required, as in the management of ovarian cancer, but also in allergy to anticancer mAbs 121.

**Table 5:** Brigham and Women's Hospital three‐bag 12‐step desensitization protocol for paclitaxel 300 mg

Target dose (mg)	300					
Standard volume per bag (ml)	250					
Final rate of infusion (ml/h)	80					
Calculated target concentration (mg/ml)	1.2					
Standard time of infusion (min)	187.5					
	**Volume**		**Concentration (mg/ml)**		**Total mg per bag**	**Amount infused (ml)**
Solution 1	250	ml of	0.012	mg/ml	3	9.38
Solution 2	250	ml of	0.120	mg/ml	30	18.75
Solution 3	250	ml of	1.190	mg/ml	297.638	250
Note: The total volume and dose dispensed are more than the final dose given to patient because many of the solutions are not completely infused						
**Step**	**Solution**	**Rate (ml/h)**	**Time (min)**	**Volume infused per step (ml)**	**Dose administered with this step (mg)**	**Cumulative dose (mg)**
1	1	2.5	15	0.63	0.0075	0.0075
2	1	5	15	1.25	0.015	0.0225
3	1	10	15	2.5	0.03	0.0525
4	1	20	15	5	0.06	0.1125
5	2	5	15	1.25	0.15	0.2625
6	2	10	15	2.5	0.3	0.5625
7	2	20	15	5	0.6	1.1625
8	2	40	15	10	1.2	2.3625
9	3	10	15	2.5	2.9764	5.3389
10	3	20	15	5	5.9528	11.2916
11	3	40	15	10	11.9055	23.1971
12	3	80	174.375	232.5	276.8029	300
Total time: 5.66 h						

Due to the increased utilization of chemotherapies and targeted mAbs hypersensitivity reactions to these medications have increased dramatically worldwide, preventing the use of first‐line therapies, with consequent impact in patient's survival and quality of life 122. These reactions can range from mild cutaneous reactions to life‐threatening symptoms including anaphylactic shock with IgE and/or mast cell/basophil involvement, and occur during or within one 1 h of the drug infusion or hours–days, as these patients have extensive premedications, including steroids 123. The symptoms are associated with the release of tryptase and other mediators such as histamine, leukotrienes and prostaglandins, implicated in the cutaneous, respiratory, gastrointestinal and cardiovascular symptoms 124. Other systemic symptoms such as chills and fever are thought to be due to the release of IL‐1 and IL‐6 among others 125. Atypical symptoms such as pain have been associated with taxanes and some monoclonal antibodies 126. Deaths have been reported when re‐exposing patients to chemotherapy drugs to which they have presented immediate hypersensitivity reactions 127. Delayed reactions can present as either mast cell/basophil‐mediated symptoms or as delayed cell‐mediated‐type intravenous (i.v.) hypersensitivity 128.

There is increasing evidence that patients with immediate and delayed hypersensitivity to chemotherapy and monoclonal antibodies can be safely re‐exposed to these medications through rapid drug desensitization (RDD) 129, in which diluted amounts of drug are reintroduced through a multistep protocol, achieving the target dose in few hours (Fig. 4B). Thereby, inhibitory mast cell mechanisms protect the patients against anaphylaxis 130. Most patients with hypersensitivity reactions are candidates for RDD, except for patients with Steven Johnsons syndrome (SJS), toxic epidermal necrolysis, drug reaction with eosinophilia and systemic symptoms (DRESS), and acute eczematous generalized pustulosis. The success of RDD relies on personalized protocols 131. Platins including carboplatin, cisplatin and oxaliplatin, taxanes including paclitaxel and docetaxel, and monoclonal antibodies such as rituximab, trastuzumab and cetuximab have been successfully desensitized 132. The largest desensitization study worldwide reported that 370 highly allergic patients received 2177 successful desensitizations to 15 drugs, three of which (bevacizumab, tocilizumab and gemcitabine) were unprecedented and in which 93% of the procedures had no or mild reactions, 7% moderate to severe reactions which did not preclude the completion of the treatment, and there were no deaths (Fig. 4C) 133. The study indicates that the overall health costs were not increased over standard treatment. Most importantly, a group of women with ovarian cancer sensitized to carboplatin had a non‐statistically significant lifespan advantage over nonallergic controls.

Therefore, IgE‐ and non‐IgE‐mediated chemotherapy hypersensitivity reactions can be managed by RDD, enabling sensitized patients to receive the full treatment safely, thus representing an important advance in the patient's treatment and prognosis.

## Conclusions

This position paper summarizes current knowledge and developments in the field of AO since 1. Novel insights gained highlight the merits of studying the nature of Th2 immune responses in cancer, much of which remains insufficiently understood. Epidemiologic analyses support associations between allergies, allergen‐specific and total IgE levels with lower risk of cancer development, to date only shown with regard to specific malignancies. Whether these associations relate to antigen‐ or allergen‐specific responses or whether they represent protective effects of IgE through recognition of specific tumour antigens remains unclear. Understanding these associations and the contributions of IgE and Th2 immunity in protection from cancer growth would also contribute to understanding whether patients with allergic asthma who receive anti‐IgE treatment may be at risk of developing cancer. Short‐term follow‐up findings have not revealed any enhanced risk of cancer development to date 134; however, further longer follow‐up studies and novel functional insights will be informative.

Emerging studies further support the study of the prototypic Th2 isotype, IgE as a means to combat tumours when directed against cancer antigens through promoting the interaction between effector and cancer cells, and stimulating CTLs via antigen cross‐presentation. Collectively, these findings support the unique properties of IgE to activate anticancer immune responses in passive and active immunotherapy of cancer and provide evidence of safety. Whereas cell‐fixed tumour antigen can trigger cross‐linking of IgE on its Fc receptors expressed on effector cells, monovalent soluble antigen does not. Some *in vivo* models relevant to IgE biology have been designed with careful consideration of species‐specific IgE receptor expression profile. Mouse models have been used most often, whereas rats, dogs and NHP may offer new alternatives to address specific questions of potency, safety and function. A couple of recombinant anticancer IgE antibodies are in the pipeline, and parallel interrogation of the same antibody immunotherapies in clinical oncology will determine the predictive value of *in vivo* models.

Recent findings shed light into the alternative Th2 antibody isotype IgG4 and its expression and functions in melanoma and other cancers 135. The mechanisms of this humoral immune bias in oncology merit further in‐depth study. Finally, it has to be emphasized that allergic reactions to anticancer agents, chemotherapy and biologics represent important challenges in daily clinical oncology practice, which can be dealt with by desensitization protocols analogous to those used in allergen immunotherapy.

In summary, the AO field represents an open interdisciplinary science forum where different aspects of the interface between allergy and cancer are systematically addressed and discussed, gaining thereby previously unappreciated insights for cancer immunotherapy.

## Author contributions

Turner MC contributed to the ‘Epidemiology’ section. Singer J, Redegeld F, Spillner E and Karagiannis P contributed to the ‘Th2‐associated antibodies in cancer’ section. Meshcheryakova A, Mechtcheriakova D and Mungenast F contributed to the ‘*In situ* expression of AID and potential insights into antibody isotype expression in cancer’ section. Untersmayr E contributed to the ‘IgE receptor expression on immune cells and epithelial cells’ section. The following authors contributed to the ‘Effector cells in allergy and cancer’ section: Levi‐Schaffer F, Mekori Y, Bax HJ and Josephs DH; Capron M and Gatault S to ‘Eosinophils’; Bianchini R and Jensen‐Jarolim E to ‘Macrophages’; Fiebiger E to ‘Dendritic cells’; and Janda J to ‘T lymphocytes’. The following authors contributed to the ‘Translational strategies to target cancer’ section: Nigro EA, Vangelista L, Siccardi AG and Jensen‐Jarolim E contributed to the ‘Tumour vaccines and adjuvants’ section; Daniels‐Wells TR, Penichet ML, Spillner E and Gould HJ to ‘Recombinant IgE anticancer antibodies’; Josephs DH, Bax HJ, Karagiannis P, Saul L and Karagiannis SN to ‘*In vitro* effector functions of IgE antibodies in the cancer context’; Dombrowicz D, Fazekas J, Bax HJ, Daniels‐Wells TR, Penichet ML, Jensen‐Jarolim E and Karagiannis SN to ‘*In vivo* models in AllergoOncology’; and Castells M, Corrigan C and Spicer JF to ‘Allergy in clinical oncology’. Jensen‐Jarolim E and Karagiannis SN wrote abstract, introduction and conclusion, contributed to several sections and compiled the whole opus; and Fazekas J designed all figures. All authors have read and approved the position paper.

## Conflicts of interest

The authors declare that they have no conflicts of interest.
